# The energy release rate for non-penetrating crack in poroelastic body by fluid-driven fracture

**DOI:** 10.1177/10812865221086547

**Published:** 2022-04-20

**Authors:** Victor A Kovtunenko, Nyurgun P Lazarev

**Affiliations:** Institute for Mathematics and Scientific Computing, University of Graz, Graz, Austria; Lavrentyev Institute of Hydrodynamics, Siberian Division of the Russian Academy of Sciences, Novosibirsk, Russia; Regional Scientific and Educational Mathematical Center “Far Eastern Center of Mathematical Research,” North-Eastern Federal University, Yakutsk, Russia

**Keywords:** Poroelasticity, hydraulic fracturing, crack, contact, incremental formulation, variational inequality, Lagrangian, asymptotic analysis, shape derivative, energy release rate, path-independent integral

## Abstract

A new class of constrained variational problems, which describe fluid-driven cracks (that are pressurized fractures created by pumping fracturing fluids), is considered within the nonlinear theory of coupled poroelastic models stated in the incremental form. The two-phase medium is constituted by solid particles and fluid-saturated pores; it contains a crack subjected to non-penetration condition between the opposite crack faces. The inequality-constrained optimization is expressed as a saddle-point problem with respect to the unknown solid phase displacement, pore pressure, and contact force. Applying the Lagrange multiplier approach and the Delfour–Zolésio theorem, the shape derivative for the corresponding Lagrangian function is derived using rigorous asymptotic methods. The resulting formula describes the energy release rate under irreversible crack perturbations, which is useful for application of the Griffith criterion of quasi-static fracture.

## 1. Introduction to poroelastic modeling

In the paper, we proceed the development of constrained optimization theory for a new class of variational models arising in poroelasticity and motivated by hydrofracking. A two-phase poroelastic body consisting of solid phase and pores saturated with a Newtonian fluid is considered. We suggest that the body contains a fluid-driven crack (called fractures) since formed by the pressure of a pumped fluid. For physical consistency, the crack is subjected to a non-penetration inequality between opposite faces (the fracture walls). This description allows a compressive pressure at which the crack might close. Here, it would be worthwhile to comment that mutual contact of adjacent crack faces admits the phenomenon of mechanically closed, but hydraulically open cracks (which could arise, e.g., through the presence of debris in an otherwise fluid-conducting crack).

The poroelastic model is described by governing equations stated in incremental form with respect to unknown solid phase displacement, pore pressure, and contact force. The system is endowed with the fluid pressure, which is prescribed inhomogeneous and different on the fracture walls. In the multi-scale formulation, the pressurized fracture equations are coupled with governing equations for the fluid pressure to a single model. Typically, fluid flow in the fracture is governed by the Reynolds lubrication equation, which assumes a local cubic law (see Baykin and Golovin [1]). Modeling of the fluid pressure using a linear diffraction equation was suggested in Mikelić et al. [2]. In our work, we account for the channelized fluid flow as a prescribed boundary condition. In its turn, the boundary data can be achieved by flow modeling as well as directly from geomechanical data.

The nonlinear theory of solids with non-penetrating cracks and their quasi-static propagation was developed in the variational framework by Khludnev and co-authors [3,4]. For dynamic modeling of cracks, we cite the monograph of Bratov et al. [5]. The non-penetration approach was continued for frictional contact phenomena at the crack in Itou et al. [6] and the limiting small strain in the proceeding works [7,8]. We cite the study [9] for Timoshenko plates with cracks, and the study [10] addressing optimal control problems. Also anti-cracks, rigid, and soft inclusions were incorporated in the theory (see Khludnev et al. [11]). For suitable numerical methods, see Hintermüller et al. [12]. Recently, in Kovtunenko [13], we derived non-penetration conditions at the fluid-driven crack in two-phase poroelastic medium.

Alternatively to the sharp-interface approach, in a brittle zone, the crack surface can be approximated by a phase-field function as described in Mikelić et al. [14] which may be beneficial for numerical reasoning. Then, the crack and its propagation are determined based on the energy minimization approach to brittle and quasi-brittle fracture (see Kovtunenko [15]). The readers may find helpful the discrete perturbation of global potentials due to crack extension in the vein of variational eigen-erosion methods from Schmidt et al. [16].

The concept of soil and poromechanics was established well by Biot and Terzaghi [17,18] and further developed by Barenblatt et al. [19] and Meirmanov [20] and others. We cite Fellner and Kovtunenko [21] and Kovtunenko and Zubkova [22] for homogenization of a two-phase medium consisted of solid phase and pores, and Sazhenkov et al. [23] for the related multi-scale analysis. In our modeling, we follow the hydraulic fracturing formulation given by Golovin and Baykin [24] and Skopintsev et al. [25] with co-authors as presented next.

For a linear elastic solid phase, the second-order symmetric tensors of linearized strain ε and Cauchy stress σ are connected by Hooke’s law

(1) $$\sigma =A\varepsilon +\tau^{0},$$

with the help of the fourth-order symmetric tensor of elastic coefficients A, which assumed to be elliptic, and subjected to a prestress $\tau^{0}$. The prestress admits mechanical stresses of geological layers in reservoir in their natural state as well by fracking (see the influence of the prestress on the failure zone development in Valov et al. [26]). Accounting for the pore pressure p, the effective stress is introduced as

(2) τ=σ−αpI,

where α∈(0,1] is the Biot coefficient, and I is the identity tensor. Omitting inertia terms in equations of motion and keeping the minus sign, the quasi-static equilibrium equation reads

(3) −divτ=0.

After substitution into equation (3) of equations (1) and (2) and the symmetric gradient of the displacement vector u

(4) $$\varepsilon \left(u\right)=\frac{1}{2}\left(\nabla u+\nabla u^{T}\right),$$

where ^T^ stands for the transposition; it implies the elliptic equation with respect to unknown u

(5) $$-d\mathrm{iv}\left(A\varepsilon \left(u\right)+\tau^{0}-\alpha pI\right)=0.$$

The fluid content in pores is constituted by

(6) ζ=Sp+αtrε,

where S>0 is the storativity, and trε implies dilatation according to equation (4). In the mass balance

(7) $$\frac{\partial \zeta }{\partial t}=-d\mathrm{iv}q,$$

the flow velocity vector q is assumed given by the Darcy flow

(8) q=−κ∇p,

where $\kappa =k_{r}/\eta_{r}$ is determined by the permeability $k_{r}>0$ and the effective viscosity $\eta_{r}>0$. Inserting equations (6) and (8) into equation (7) results in the parabolic equation with respect to ∂p/∂t and ∂u/∂t

(9) $$\frac{\partial }{\partial t}\left(\mathrm{Sp}+\alpha tr\varepsilon \left(u\right)\right)-d\mathrm{iv}\left(\kappa \nabla p\right)=0.$$

From the mathematical point of view, the fully coupled poroelastic equations (5) and (9) present a degenerate elliptic-parabolic system; thus, standard existence theorems are not applicable here. After differentiation of the elliptic equation (5) with respect to time, the system turns into a pure parabolic problem. Its solvability was established by applying the theory of implicit evolution equations (see Showalter [27]). However, the parabolic problem does not conform to the unilateral conditions. On the other side, the governing equations formally coincide with thermoelastic equations when replacing the pore pressure p for temperature. From the literature on thermoelasticity, existence results utilizing the pseudo-monotonone theory were known (see Khludnev and Kovtunenko [3], section 3.3), however, restricted to small coupling coefficients α. Avoiding these restrictive assumptions, in Kovtunenko [13] we proved the well-posedness based on Rothe’s semi-discretization in time of parabolic equation (9), which reduces it to the elliptic equation with respect to unknown p at fixed time t>δ>0

(10) $$S\left(p-p_{t-\delta }\right)+\alpha tr\varepsilon \left(u-u_{t-\delta }\right)-\delta d\mathrm{iv}\left(\kappa \nabla p\right)=0,$$

for given $p_{t-\delta }:=p(t-\delta )$ and $u_{t-\delta }:=u(t-\delta )$, then passing the time step δ to zero.

In the current contribution, we investigate shape differentiability of the poroelastic problem with non-penetrating crack under irreversible shape perturbations. For this task, we consider the problem in the incremental form (5) and (10), and endow it with a saddle-point formulation. Based on the Lagrange multiplier approach, we apply the formalism of directional differentiability for Lagrangians (see Delfour and Zolésio [28]) and use rigorous asymptotic methods (see González et al. [29]) to derive a shape derivative for the underlying Lagrangian function implying a free energy. The resulting formula describes the energy release rate under irreversible crack perturbations, which is useful for the Griffith criterion of quasi-static crack evolution (see Charlotte et al. [30]). Other shape derivatives were derived in a series of works for non-penetrating cracks and inclusions in linear elastic bodies by Khludnev and his colleagues [3,4] in Khludnev and Shcherbakov [31] within the Euler–Bernoulli beam theory, in Rudoy and Shcherbakov [32] for Kirchhoff–Love plates, in Lazarev [33], Lazarev and Rudoy [34] for Timoshenko plates, and so on.

The structure of the paper is the following one. In section 2, we state the poroelastic problem with non-penetrating crack in the incremental form. In section 3, variational principle is given for a Lagrangian function, and well-posedness of the corresponding saddle-point problem is established. Also, we formulate the Griffith fracture criterion for the crack quasi-static evolution. In section 4, the shape differentiability of the Lagrangian is proven using the asymptotic methods of analysis based on regular perturbations, thus providing us with a semi-analytic formula for the energy release rate. Special cases of the formula are discussed in section 5; its relation to well-known path-independent integrals (called Cherepanov–Rice, or J-integrals) is presented.

## 2. Formulation of poroelastic problem with non-penetrating crack

**Figure 1.**
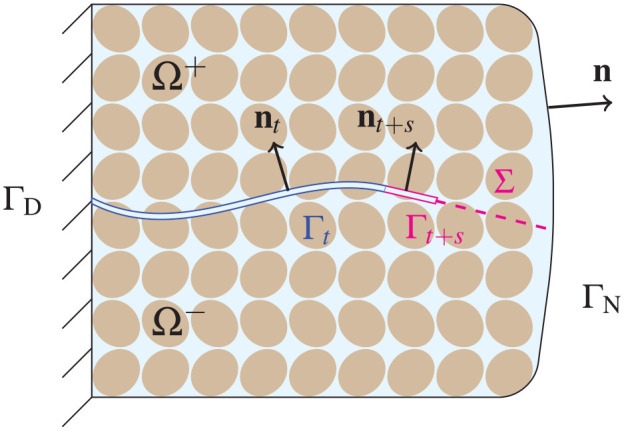
Example geometry of a poroelastic body with evolving crack in 2D.

We start with a description of geometry as illustrated for 2D in Figure 1.

Let Ω be a reference domain in the Euclidean space of points $x=(x_{1},\ldots ,x_{d}{)}^{T}\in R^{d}$, d=2,3. We assume the Lipschitz continuous boundary ∂Ω with outward normal vector $n=(n_{1},\ldots ,n_{d}{)}^{T}$, and the disjoint union $\partial \Omega =\Gamma_{D}\overset{\cdot }{⋃}\Gamma_{N}$ with $\Gamma_{D}\neq Ø$. Let an oriented manifold of co-dimension one Σ split Ω into two sub-domains $\Omega^{\pm }$ with Lipschitz continuous boundaries $\partial \Omega^{\pm }$ such that

(11) $$\partial \Omega^{+}\cap \partial \Omega^{-}=\Sigma ,\;\Omega =\Omega^{+}\cup \Omega^{-}\cup \Sigma ,\;\partial \Omega^{\pm }\cap \Gamma_{D}\neq Ø.$$

For a time parameter t∈(0,T], T>0, we look for a crack evolution along the interface

(12) $$t\mapsto \Gamma_{t}\subset \Sigma ,$$

which is assumed to be $C^{1,1}$-smooth and irreversible such that

(13) $$\Gamma_{t}\subset \Gamma_{t+s}\;\forall s\in \left(0,T-t\right).$$

We distinguish the crack faces $\Gamma_{t}^{\pm }\subset \Sigma^{\pm }$ and chose the normal vector $n_{t}$ at $\Gamma_{t}$ outward to $\Omega^{-}$, thus inward to $\Omega^{+}$. Physically, $\Gamma_{t}$ represents fractures, whereas the complement

(14) $$\Omega_{t}:=\Omega \backslash \bar{\Gamma_{t}},$$

implies a reservoir.

For every fixed t∈(0,T] and $x\in \Omega_{t}$ in the time-dependent domain from equations (11) to (14), the poroelastic medium is described by the pore pressure p(t,x) and the solid displacement $u=(u_{1},\ldots ,u_{d}{)}^{T}(t,x)$. The latter is involved in the strain $\varepsilon (u)=\{\varepsilon_{\mathrm{ij}}(u){\}}_{i,j=1}^{d}$ according to equation (4) with the entries $u_{i,j}:=\partial u_{i}/\partial x_{j}$ of the gradient $\nabla u=\{u_{i,j}{\}}_{i,j=1}^{d}$. The stress $\sigma =\{\sigma_{\mathrm{ij}}{\}}_{i,j=1}^{d}(t,x)$ and the effective stress $\tau =\{\tau_{\mathrm{ij}}{\}}_{i,j=1}^{d}(t,x)$ are introduced in equations (1) and (2), respectively, and the prestress is given by the symmetric tensor

$$\tau^{0}={\left\{\tau_{\mathrm{ij}}^{0}\right\}}_{i,j=1}^{d}\left(x\right)\in H^{1}{\left(\Omega \right)}_{\mathrm{sym}}^{d\times d}:=H^{1}\left(\Omega ;R_{s\mathrm{ym}}^{d\times d}\right).$$

For the reason of analysis, we do not consider here the so-called 2.5D models when physical strain and stress are 3×3-tensors defined over a 2D-domain Ω. The system is governed by equations (5) and (10), where componentwisely $(d\mathrm{iv}\;\tau {)}_{i}:=\sum_{j=1}^{d}\tau_{\mathrm{ij},j}$ for i=1,…,d, and the trace $tr\;\varepsilon (u)=d\mathrm{iv}\;u:=\sum_{i=1}^{d}u_{i,i}$. The elastic coefficients in equation (5)

$$A={\left\{A_{\mathrm{ijkl}}\right\}}_{i,j,k,l=1}^{d}\left(x\right)\in W^{1,\infty }{\left(\Omega \right)}_{s\mathrm{ym}}^{d\times d\times d\times d},$$

are symmetric: $A_{\mathrm{ijkl}}=A_{\mathrm{jikl}}=A_{\mathrm{klij}}$ for i,j,k,l=1,…,d, and build a self-adjoint bilinear form

(15) $$\int_{\Omega_{t}}A\varepsilon \left(u\right):\varepsilon \left(v\right)dx:=\sum_{i,j,k,l=1}^{d}\int_{\Omega_{t}}A_{\mathrm{ijkl}}\varepsilon_{\mathrm{kl}}\left(u\right)\varepsilon_{\mathrm{ij}}\left(v\right)dx,$$

for all $u,v\in H^{1}(\Omega_{t}{)}^{d}$, which is uniformly elliptic and bounded: there exists $0<\underline{a}\leq \bar{a}$ such that

(16) $$\underline{a}∥u{∥}_{H^{1}(\Omega_{t})}^{2}\leq \int_{\Omega_{t}}A\varepsilon \left(u\right):\varepsilon \left(u\right)dx,\;\left|\int_{\Omega_{t}}A\varepsilon \left(u\right):\varepsilon \left(v\right)dx\right|\leq \bar{a}∥u{∥}_{H^{1}(\Omega_{t})}∥v{∥}_{H^{1}(\Omega_{t})},$$

holds for all t∈[0,T] according to Korn and Poincaré inequalities if u=0 on $\Gamma_{D}$. In equation (10), the transport coefficient $\kappa \in W^{1,\infty }(\Omega )$ is assumed uniformly positive and bounded

(17) $$0<\underline{\kappa }\leq \kappa \left(x\right)\leq \bar{\kappa },$$

and the time-delayed data in $\Omega_{t-\delta }$ for t∈(δ,T) are given by functions

$$u_{t-\delta }={\left({\left(u_{t-\delta }\right)}_{1},\ldots ,{\left(u_{t-\delta }\right)}_{d}\right)}^{T}\left(t,x\right)\in H^{2}{\left(\Omega_{t-\delta }\right)}^{d},\;p_{t-\delta }\left(t,x\right)\in H^{2}\left(\Omega_{t-\delta }\right),$$

such that the irreversibility of crack evolutions (13) provides the inclusion $H^{2}(\Omega_{t-\delta })\subset H^{2}(\Omega_{t})$ , hence

(18) $$u_{t-\delta }\in H^{2}{\left(\Omega_{t}\right)}^{d},\;p_{t-\delta }\in H^{2}\left(\Omega_{t}\right)\;\mathrm{for}\;\mathrm{all}\;t\in \left(\delta ,T\right).$$

We decompose the displacement u and the stress $\tau n:=(\sum_{j=1}^{d}\tau_{1j}n_{j},\ldots ,\sum_{j=1}^{d}\tau_{\mathrm{dj}}n_{j}{)}^{T}$ at the boundary into its normal components

$$n^{T}u:=\sum_{i=1}^{d}n_{i}u_{i},\;n^{T}\tau n=\sum_{i,j=1}^{d}n_{i}\tau_{\mathrm{ij}}n_{j},$$

implying the vector–vector and matrix–vector multiplications, and tangential components as follows

$$u=\left(n^{T}u\right)n+\left(u-\left(n^{T}u\right)n\right),\;\tau n=\left(n^{T}\tau n\right)n+\left(\tau n-\left(n^{T}\tau n\right)n\right).$$

Let the following time-dependent data are prescribed in the reservoir for all t∈(0,T)

(19) $$g={\left(g_{1},\ldots ,g_{d}\right)}^{T}\left(t,x\right)\in L^{2}{\left(\Gamma_{N}\right)}^{d},\;p_{re}\left(t,x\right)\in H^{2}\left(\Omega_{t}\right).$$

With its help, we state mixed inhomogeneous boundary conditions on the outer boundary

(20) $$u=0\;\mathrm{on}\;\Gamma_{D},\;\tau n=g\;\mathrm{on}\;\Gamma_{N},\;p=p_{re}\;\mathrm{on}\;\partial \Omega .$$

The assumed regularity of the data will be used further for asymptotic expansions in sections 4 and 5.

Across the crack $\Gamma_{t}$, functions defined in $\Omega_{t}$ allow discontinuity by the mean of jump

$$\left[\left[u\right]\right]:=u{|}_{\Gamma_{t}^{+}}-u{|}_{\Gamma_{t}^{-}},\;\left[\left[\tau \right]\right]:=\tau {|}_{\Gamma_{t}^{+}}-\tau {|}_{\Gamma_{t}^{-}},\;\left[\left[p\right]\right]:=p{|}_{\Gamma_{t}^{+}}-p{|}_{\Gamma_{t}^{-}}.$$

We suggest no tangential effective stress at the crack faces

(21) $$\tau n_{t}-\left(n_{t}^{T}\tau n_{t}\right)n_{t}=0\;\mathrm{on}\;\Gamma_{t}^{\pm },$$

and continuity of the fluid pressure over the fracture walls

(22) $$p=p_{re}^{\pm }\;\mathrm{on}\;\Gamma_{t}^{\pm }.$$

The fluid pressure $p_{re}^{\pm }$ prescribed in equation (19) is different on $\Gamma_{t}^{\pm }$, coincide at the crack-tip, respectively, crack-front in 3D, and can be determined from the lubrication equations in fractures (see Golovin and Baykin [24]).

Assuming at $\Gamma_{t}^{\pm }$ the standard boundary condition in the normal direction

(23) $$n_{t}^{T}\tau n_{t}+p_{re}^{\pm }=0,$$

would lead to interpenetration between the opposite crack faces under compressive stress. For the physical consistency, non-penetration at the crack is suggested

(24) $$n_{t}^{T}\left[\left[u\right]\right]\geq 0\;\mathrm{on}\;\Gamma_{t},$$

see Figure 1. The inequality constraint (24) leads to complementary conditions

(25) $$\left[\left[n_{t}^{T}\tau n_{t}+p_{re}\right]\right]=0,\;n_{t}^{T}\tau n_{t}+p_{re}\leq 0,\;\left(n_{t}^{T}\tau n_{t}+p_{re}\right)\left(n_{t}^{T}\left[\left[u\right]\right]\right)=0\;\mathrm{on}\;\Gamma_{t}.$$

Conditions (25) imply that equality (23) holds at those points where the crack is open, i.e., $n_{t}^{T}[[u]]>0$. Otherwise, the closed crack $n_{t}^{T}[[u]]=0$ in equation (25) has the compressive stress $n_{t}^{T}\tau n_{t}+p_{\mathrm{re}}\leq 0$.

## 3. Variational principle for the crack problem

In the domain with crack defined in equation (14), we have the following generalized Green’s formula (see Khludnev and Kovtunenko [3], section 1.4) for the elasticity operator

(26) $$-\int_{\Omega_{t}}{\left(d\mathrm{iv}\tau \right)}^{T}vdx=\int_{\Omega_{t}}\tau :\varepsilon \left(v\right)dx-{〈\tau n,v〉}_{\Gamma_{N}}+{〈\tau n_{t},v〉}_{\Gamma_{t}^{+}}-{〈\tau n_{t},v〉}_{\Gamma_{t}^{-}},$$

for all $\tau \in L^{2}(\Omega_{t}{)}_{s\mathrm{ym}}^{d\times d}$ and $d\mathrm{iv}\tau \in L^{2}(\Omega_{t}{)}^{d},v\in H^{1}(\Omega_{t}{)}^{d}$ with v=0 on $\Gamma_{D}$. Here, the boundary stresses τn on $\Gamma_{N}$ and $\tau n_{t}$ on $\Gamma_{t}^{\pm }$ are distributions defined in a generalized sense by duality mappings $〈\cdot ,\cdot {〉}_{\Gamma_{N}}$ and $〈\cdot ,\cdot {〉}_{\Gamma_{t}^{\pm }}$, which turn into usual integrals for functions. For the stationary transport operator, Green’s formula

(27) $$-\int_{\Omega_{t}}d\mathrm{iv}\left(\kappa \nabla p\right)qdx=\int_{\Omega_{t}}\kappa \nabla p^{T}\nabla qdx,$$

holds for all functions $p\in H^{1}(\Omega_{t}),\Delta p\in L^{2}(\Omega_{t})$, and $q\in H_{0}^{1}(\Omega_{t})$.

Inserting into equation (26) the equilibrium equation (5), using the Neumann condition τn=g on $\Gamma_{N}$ from equation (20), and $\tau n_{t}=(n_{t}^{T}\tau n_{t})n_{t}$ at the crack due to the zero tangential stress in equation (21), we obtain

$$0=\int_{\Omega_{t}}\tau :\varepsilon \left(v\right)dx-\int_{\Gamma_{N}}g^{T}vdS_{x}+{〈n_{t}^{T}\tau n_{t},n_{t}^{T}v〉}_{\Gamma_{t}^{+}}-{〈n_{t}^{T}\tau n_{t},n_{t}^{T}v〉}_{\Gamma_{t}^{-}}.$$

By the virtue of $[[n_{t}^{T}\tau n_{t}+p_{re}]]=0$ in equation (25), adding and subtracting $p_{re}$ follows the variational equation with respect to τ

(28) $$\int_{\Omega_{t}}\tau \;:\varepsilon \left(v\right)dx-\int_{\Gamma_{N}}g^{T}vdS_{x}-\int_{\Gamma_{t}}n_{t}^{T}\left[\left[p_{re}v\right]\right]dS_{x}+{〈n_{t}^{T}\tau \;n_{t}+p_{re},n_{t}^{T}\left[\left[v\right]\right]〉}_{\Gamma_{t}}=0,$$

for all test functions $v\in H^{1}(\Omega_{t}{)}^{d}$ with v=0 on $\Gamma_{D}$. The jump at the crack $\Gamma_{t}$ is well defined

$$n_{t}^{T}\left[\left[v\right]\right]\in H_{00}^{1/2}\left(\Gamma_{t}\right),$$

in the Lions–Magenes space of functions, which continuation by zero in Σ belongs to $H^{1/2}(\Sigma )$. Its counter-part in the duality $〈\cdot ,\cdot {〉}_{\Gamma_{t}}$ is determined in the adjoint space of linear continuous functionals

(29) $$\lambda :=n_{t}^{T}\tau n_{t}+p_{re}\in H_{00}^{1/2}{\left(\Gamma_{t}\right)}^{★}.$$

Then, the complementarity conditions (24) and (25) take the weak form

(30) $$n_{t}^{T}\left[\left[u\right]\right]\geq 0,\;{〈\lambda ,\xi -n_{t}^{T}\left[\left[u\right]\right]〉}_{\Gamma_{t}}\leq 0\;\mathrm{for}\;\mathrm{all}\;\xi \in H_{00}^{1/2}\left(\Gamma_{t}\right)\;\mathrm{such}\;\mathrm{that}\;\xi \geq 0,$$

and newly introduced variable λ in equation (29) implies the contact force. Inserting the transport equation (10) into Green’s formula (27) and using equation (18) results in the variational equation with respect to p

(31) $$\int_{\Omega_{t}}\left(\left[S\left(p-p_{t-\delta }\right)+\alpha tr\;\varepsilon \left(u-u_{t-\delta }\right)\right]q+\delta \kappa \nabla p^{T}\nabla q\right)dx=0,$$

for all test functions $q\in H_{0}^{1}(\Omega_{t})$.

Gathering the weak variational formulation (28)–(31) and recalling $\tau \;=A\varepsilon (u)+\tau^{0}-\alpha pI$, for the triple (u,p,λ), we define a Lagrange function $L:H^{1}(\Omega_{t}{)}^{d}\times H^{1}(\Omega_{t})\times H_{00}^{1/2}(\Gamma_{t}{)}^{★}\mapsto R$ by

(32) $$\begin{array}{c} L\left(u,p,\lambda ;\Gamma_{t}\right):=\int_{\Omega_{t}}\left\{\left(\frac{1}{2}A\varepsilon \left(u\right)+\tau^{0}\right):\varepsilon \left(u\right)-\left[S\left(\frac{1}{2}p-p_{t-\delta }\right)+\alpha tr\;\varepsilon \left(u-u_{t-\delta }\right)\right]p-\frac{\delta }{2}\kappa |\nabla p{|}^{2}\right\}dx\\ \;-\int_{\Gamma_{N}}g^{T}udS_{x}-\int_{\Gamma_{t}}n_{t}^{T}\left[\left[p_{re}u\right]\right]dS_{x}+{〈\lambda ,n_{t}^{T}\left[\left[u\right]\right]〉}_{\Gamma_{t}}, \end{array}$$

accounting for the identity αpI:ε(u)=αtrε(u)p and multiplying the quadratic terms by 1/2. With its help existence of a weak solution to the problem is established in the next.

### Theorem 1 (solution existence)

There exists a triple $\left(u_{t},p_{t}-p_{re},\lambda_{t})\in K(\Omega_{t}\right)$ in the feasible set

$$K\left(\Omega_{t}\right):=\left\{\left(v,q,\mu \right)\in H^{1}{\left(\Omega_{t}\right)}^{d}\times H_{0}^{1}\left(\Omega_{t}\right)\times H_{00}^{1/2}{\left(\Gamma_{t}\right)}^{★}|\;v=0\;\mathrm{on}\;\Gamma_{D}\;\mathrm{and}\;\mu \leq 0\right\},$$

solving uniquely the saddle-point problem

(33) $$L\left(u_{t},q,\mu ;\Gamma_{t}\right)\leq L\left(u_{t},p_{t},\lambda_{t};\Gamma_{t}\right)\leq L\left(v,p_{t},\lambda_{t};\Gamma_{t}\right),$$

for all test functions $\left(v,q,\mu )\in K(\Omega_{t}\right)$. Then, it solves the poroelastic problem with non-penetrating crack stated in the weak form of equations (28)–(31), and vice versa.

### Proof

With respect to the primal variable $u\mapsto L(u,p,\lambda ;\Gamma_{t})$, the Lagrangian in equation (32) builds a quadratic bilinear form, which is bounded and positive definite due to the estimates (16) of A. With respect to the dual variable, the quadratic bilinear form $p\mapsto L(u,p,\lambda ;\Gamma_{t})$ is bounded and negative definite because of estimates (17) of κ. The mapping $\lambda \mapsto L(u,p,\lambda ;\Gamma_{t})$ is linear. Therefore, the unique saddle-point in equation (33) exists by the virtue of minimax theorems.

Based on the optimality condition for equation (33), we calculate the Gateaux derivative of the Lagrangian

$$\underset{s\to 0}{\lim}\frac{1}{s}\left(L\left(u_{t}+sv,p_{t},\lambda_{t};\Gamma_{t}\right)-L\left(u_{t},p_{t},\lambda_{t};\Gamma_{t}\right)\right)=0,$$

and get the variational equation (28) for $u=u_{t}$, the stress $\tau_{t}:=A\varepsilon (u_{t})+\tau^{0}-\alpha p_{t}I$, and the contact force $n_{t}^{T}\tau_{t}n_{t}+p_{re}=\lambda_{t}$ according to equation (29). Conversely, from equation (28), it follows by convexity the minimum in equation (33)

$$L\left(u_{t},p_{t},\lambda_{t};\Gamma_{t}\right)\leq L\left(v,p_{t},\lambda_{t};\Gamma_{t}\right).$$

Similarly, computing the limit

$$\underset{s\to 0}{\lim}\frac{1}{s}\left(L\left(u_{t},p_{t}+\mathrm{sq},\lambda_{t};\Gamma_{t}\right)-L\left(u_{t},p_{t},\lambda_{t};\Gamma_{t}\right)\right)=0,$$

results in equation (31) for $p=p_{t}$ and $u=u_{t}$. The converse assertion that equation (31) implies the maximum

$$L\left(u_{t},q,\lambda_{t};\Gamma_{t}\right)\leq L\left(u_{t},p_{t},\lambda_{t};\Gamma_{t}\right),$$

is true by the concavity of $p\mapsto L(u,p,\lambda ;\Gamma_{t})$. The maximum in equation (33) with respect to μ taken at $q=p_{t}$ implies the dual complementarity conditions

(34) $$\lambda_{t}\leq 0,\;{〈\mu -\lambda_{t},n_{t}^{T}\left[\left[u_{t}\right]\right]〉}_{\Gamma_{t}}\leq 0\;\mathrm{for}\;\mathrm{all}\;\mu \in H_{00}^{1/2}{\left(\Gamma_{t}\right)}^{★}\mathrm{such}\;\mathrm{that}\;\mu \leq 0,$$

which are equivalent to equation (30) for $\lambda =\lambda_{t}$ and $u=u_{t}$. The proof is complete.

For a perturbation parameter s∈(0,T−t), we consider an irreversible crack perturbation $\Gamma_{t+s}$ satisfying equation (13) (see illustration in Figure 1) and the perturbed domain with crack

(35) $$\Omega_{t+s}:=\Omega \backslash \bar{\Gamma_{t+s}},$$

according to equation (14). Let space points $y=(y_{1},\ldots ,y_{d}{)}^{T}$ be related to the perturbed geometry $\Omega_{t+s}$. The perturbed Lagrangian $L:H^{1}(\Omega_{t+s}{)}^{d}\times H^{1}(\Omega_{t+s})\times H_{00}^{1/2}(\Gamma_{t+s}{)}^{★}\mapsto R$ is defined according to equation (32) as

(36) $$\begin{array}{c} L\left(v,q,\mu ;\Gamma_{t+s}\right):=\int_{\Omega_{t+s}}\left\{\left(\frac{1}{2}A\varepsilon \left(v\right)+\tau^{0}\right):\varepsilon \left(v\right)-\left[S\left(\frac{1}{2}q-p_{t-\delta }\right)+\alpha tr\;\varepsilon \left(v-u_{t-\delta }\right)\right]q-\frac{\delta }{2}\kappa |\nabla q{|}^{2}\right\}dy\\ \;-\int_{\Gamma_{N}}g^{T}vdS_{y}-\int_{\Gamma_{t+s}}n_{t+s}^{T}\left[\left[\;p_{re}v\right]\right]dS_{y}+{〈\mu ,n_{t+s}^{T}\left[\left[v\right]\right]〉}_{\Gamma_{t+s}}. \end{array}$$

The perturbed saddle-point problem (33) reads

(37) $$L\left(u_{t+s},q,\mu ;\Gamma_{t+s}\right)\leq L\left(u_{t+s},p_{t+s},\lambda_{t+s};\Gamma_{t+s}\right)\leq L\left(v,p_{t+s},\lambda_{t+s};\Gamma_{t+s}\right),$$

for all test functions $\left(v,q,\mu )\in K(\Omega_{t+s}\right)$ in the perturbed feasible set

$$K\left(\Omega_{t+s}\right)=\left\{\left(v,q,\mu \right)\in H^{1}{\left(\Omega_{t+s}\right)}^{d}\times H_{0}^{1}\left(\Omega_{t+s}\right)\times H_{00}^{1/2}{\left(\Gamma_{t+s}\right)}^{★}|\;v=0\;\mathrm{on}\;\Gamma_{D}\;\mathrm{and}\;\mu \leq 0\right\}.$$

According to Theorem 1, there exists the unique solution $\left(u_{t+s},p_{t+s}-p_{\mathrm{re}}{|}_{t+s},\lambda_{t+s})\in K(\Omega_{t+s}\right)$ to equation (37). It is also the solution to the perturbed poroelastic problem with non-penetrating crack from equations (28)–(31)

(38) $$\int_{\Omega_{t+s}}\tau_{t+s}:\varepsilon \left(v\right)dy-\int_{\Gamma_{N}}g^{T}vdS_{y}-\int_{\Gamma_{t+s}}n_{t+s}^{T}\left[\left[p_{re}v\right]\right]dS_{y}+{〈\lambda_{t+s},n_{t+s}^{T}\left[\left[v\right]\right]〉}_{\Gamma_{t+s}}=0,$$

for all test functions $v\in H^{1}(\Omega_{t+s}{)}^{d}$ with v=0 on $\Gamma_{D}$, where $\tau_{t+s}=A\varepsilon (u_{t+s})+\tau^{0}-\alpha p_{t+s}I$ and $\lambda_{t+s}=n_{t+s}^{T}\tau_{t+s}n_{t+s}+p_{re}$; the perturbed complementarity conditions

(39) $$n_{t+s}^{T}\left[\left[u_{t+s}\right]\right]\geq 0,\;{〈\lambda_{t+s},\xi -n_{t+s}^{T}\left[\left[u_{t+s}\right]\right]〉}_{\Gamma_{t+s}}\leq 0\;\mathrm{for}\;\mathrm{all}\;\xi \in H_{00}^{1/2}\left(\Gamma_{t+s}\right)\;\mathrm{such}\;\mathrm{that}\;\xi \geq 0;$$

and the perturbed stationary transport equation

(40) $$\int_{\Omega_{t+s}}\left(\left[S\left(p_{t+s}-p_{t-\delta }\right)+\alpha tr\;\varepsilon \left(u_{t+s}-u_{t-\delta }\right)\right]q+\delta \kappa \nabla p_{t+s}^{T}\nabla q\right)dy=0,$$

for all test functions $q\in H_{0}^{1}(\Omega_{t+s})$.

With the help of reduced Lagrangian L in equation (32) calculated on the saddle-point from equation (33), and its perturbation in equation (36) calculated on the saddle-point from equation (37), we define a directional derivative (called the shape derivative) as the one-sided limit

(41) $$\frac{\partial }{\partial t}L\left(u_{t},p_{t},\lambda_{t};\Gamma_{t}\right):=\underset{s\to 0^{+}}{\lim}\frac{1}{s}\left(L\left(u_{t+s},p_{t+s},\lambda_{t+s};\Gamma_{t+s}\right)-L\left(u_{t},p_{t},\lambda_{t};\Gamma_{t}\right)\right).$$

Physically, equation (41) implies the energy release rate by extension of the crack. For a constant surface energy density γ>0, let us denote the increase in surface energy due to creation of the new crack by

(42) $$G_{t}:=\underset{s\to 0^{+}}{\lim}\frac{1}{s}\left(2\gamma \int_{\Gamma_{t+s}}dS_{y}-2\gamma \int_{\Gamma_{t}}dS_{x}\right)>0.$$

Based on equations (41) and (42), Griffith’s fracture criterion can be stated as the following condition

(43) $$|\Gamma_{t+s}|\geq |\Gamma_{t}|,\;\left(\frac{\partial }{\partial t}L\left(u_{t},p_{t},\lambda_{t};\Gamma_{t}\right)+G_{t}\right)\left(|\Gamma_{t+s}|-|\Gamma_{t}|\right)\geq 0.$$

Together with irreversibility (13), the inequalities in equation (43) imply the two cases:

- if $\partial /\partial tL(u_{t},p_{t},\lambda_{t};\Gamma_{t})+G_{t}<0$, then $\Gamma_{t+s}=\Gamma_{t}$ and crack does not grow;
- if $\partial /\partial tL(u_{t},p_{t},\lambda_{t};\Gamma_{t})+G_{t}\geq 0$, then $\left|\Gamma_{t+s}|>|\Gamma_{t}\right|$ and crack will begin to grow.

For the reason of fracture criterion (43), the main aim of our further consideration will be to provide a formula for calculating the shape derivative ∂L/∂t in equation (41) (also the limit $G_{t}$ in equation (42)).

## 4. Energy release rate by fluid-driven fracture

The crack perturbation can be carried out either in explicit or implicit form. In the explicit case, given a kinematic flow

(44) $$\left[\left(s,x\right)\mapsto \phi_{s}={\left({\left(\phi_{s}\right)}_{1},\ldots ,{\left(\phi_{s}\right)}_{d}\right)}^{T},\;\phi_{s}^{-1}={\left({\left(\phi_{s}^{-1}\right)}_{1},\ldots ,{\left(\phi_{s}^{-1}\right)}_{d}\right)}^{T}\right]\in C^{1}\left(\left[0,T\right];W^{1,\infty }{\left(\Omega \right)}^{d}\right),$$

associates a coordinate transformation $y=\phi_{s}(x)$ and its inverse $x=\phi_{s}^{-1}(y)$ such that

$$\left[\phi_{s}^{-1}{}^{\circ}\phi_{s}\right]\left(x\right)=x,\;\left[\phi_{s}{}^{\circ}\phi_{s}^{-1}\right]\left(y\right)=y.$$

We suppose that it builds a diffeomorphism of the cracked domains in equations (14) and (35)

(45) $$\phi_{s}:\Omega_{t}\mapsto \Omega_{t+s},\;x\mapsto y;\;\phi_{s}^{-1}:\Omega_{t+s}\mapsto \Omega_{t},\;y\mapsto x.$$

From equation (44), a time-dependent kinematic velocity is defined as $\Lambda {|}_{t+s}:=[d\phi_{s}/\mathrm{ds}]{}^{\circ}\phi_{s}^{-1}$.

In the implicit case, let a vector of kinematic velocity

(46) $$\Lambda ={\left(\Lambda_{1},\ldots ,\Lambda_{d}\right)}^{T}\left(t,x\right)\in C\left(\left[0,T\right];W^{1,\infty }{\left(\Omega \right)}^{d}\right),$$

be given such that

(47) $$\Lambda =0\;\mathrm{on}\;\partial \Omega ,\;\Lambda^{T}n_{t}=0\;\mathrm{on}\;\Gamma_{t},$$

preserving the outer boundary and irreversible cracks in equation (13). This determines the flow in equation (45) by means of solutions to the Cauchy problem for non-autonomous and nonlinear ordinary differential equation (ODE) system

(48) $$\frac{d}{\mathrm{ds}}\phi_{s}=\Lambda \left(t+s,\phi_{s}\right)\;\mathrm{for}\;s\in \left(0,T-t\right),\;\phi_{s}=x\;\mathrm{as}\;s=0,$$

and to the initial problem for a linear transport equation

(49) $$\frac{\partial }{\partial s}\phi_{s}^{-1}+\left(\nabla_{y}\phi_{s}^{-1}\right)\Lambda {|}_{t+s}=0\;\mathrm{for}\;\left(s,y\right)\in \left(0,T-t\right)\times \Omega ,\;\phi_{s}^{-1}=y\;\mathrm{as}\;s=0,$$

where the gradient $\nabla_{y}\phi_{s}^{-1}=(\partial (\phi_{s}^{-1}{)}_{i}/\partial y_{j}{)}_{i,j=1}^{d}$, and $\Lambda {|}_{t+s}(y)=\Lambda (t+s,y)$. We assume the both equations (44) and (46) hold.

The following Traits 1–4 are needed to prove the shape differentiability of the Lagrangian.

### Trait 1 (bijection of feasible sets)

The function composition with $\phi_{s}$ forms is a bijective map between the feasible sets

(50) $$\left(v,q,\mu \right)\mapsto \left(\tilde{v},\tilde{q},\tilde{\mu }\right):=\left(v{}^{\circ}\phi_{s},q{}^{\circ}\phi_{s},\mu {}^{\circ}\phi_{s}\right):K\left(\Omega_{t+s}\right)\mapsto K\left(\Omega_{t}\right).$$

Indeed, equation (50) follows straightforwardly from the diffeomorphism in equation (45). Trait 1 allows us to transform one-to-one the perturbed Lagrangian L from equation (36) to the reference geometry by setting

(51) $$\tilde{L}\left(s,\tilde{v},\tilde{q},\tilde{\mu };\Gamma_{t}\right):=L\left(v,q,\mu ;\Gamma_{t+s}\right),\;\tilde{L}\left(0,\tilde{v},\tilde{q},\tilde{\mu };\Gamma_{t}\right)=L\left(\tilde{v},\tilde{q},\tilde{\mu };\Gamma_{t}\right),$$

for all $\left(v,q,\mu )\in K(\Omega_{t+s}\right)$. Applying equation (51) to the perturbed saddle-point problem (37), we have

(52) $$\tilde{L}\left(s,{\tilde{u}}_{t+s},\tilde{q},\tilde{\mu };\Gamma_{t}\right)\leq \tilde{L}\left(s,{\tilde{u}}_{t+s},{\tilde{p}}_{t+s},{\tilde{\lambda }}_{t+s};\Gamma_{t}\right)\leq \tilde{L}\left(s,\tilde{v},{\tilde{p}}_{t+s},{\tilde{\lambda }}_{t+s};\Gamma_{t}\right),$$

for all test functions $\left(\tilde{v},\tilde{q},\tilde{\mu })\in K(\Omega_{t}\right)$, and $\left({\tilde{u}}_{t+s},{\tilde{p}}_{t+s}-p_{re},{\tilde{\lambda }}_{t+s})\in K(\Omega_{t}\right)$ is the unique solution to equation (52). Thus, the next trait holds.

### Trait 2 (existence of saddle point)

The set of saddle-points $\left({\tilde{u}}_{t+s},{\tilde{p}}_{t+s}-p_{re},{\tilde{\lambda }}_{t+s}\right)$ in equation (52) is a singleton for every s∈[0,T−t].

We write the s-dependent Lagrangian $\tilde{L}$ defined in equation (51) in the explicit form following from equation (36)

(53) $$\begin{array}{c} \tilde{L}\left(s,\tilde{v},\tilde{q},\tilde{\mu };\Gamma_{t}\right)=\int_{\Omega_{t}}\{\left(\frac{1}{2}\tilde{A}E\left(\nabla {\tilde{\phi }}_{s}^{-T},\tilde{v}\right)+{\tilde{\tau }}^{0}\right):E\left(\nabla {\tilde{\phi }}_{s}^{-T},\tilde{v}\right)\\ \;-\left[S\left(\frac{1}{2}\tilde{q}-{\tilde{p}}_{t-\delta }\right)+\alpha tr\;E\left(\nabla {\tilde{\phi }}_{s}^{-T},\tilde{v}-{\tilde{u}}_{t-\delta }\right)\right]\tilde{q}-\frac{\delta }{2}\tilde{\kappa }|\nabla {\tilde{\phi }}_{s}^{-T}\nabla \tilde{q}{|}^{2}\}J_{s}dx\\ \;-\int_{\Gamma_{N}}g^{T}\tilde{v}dS_{x}-\int_{\Gamma_{t}}{\tilde{n}}_{t+s}^{T}\left[\left[{\tilde{p}}_{\mathrm{re}}\tilde{v}\right]\right]\omega_{s}dS_{x}+{〈\tilde{\mu },{\tilde{n}}_{t+s}^{T}\left[\left[\tilde{v}\right]\right]\omega_{s}〉}_{\Gamma_{t}}. \end{array}$$

Here, we have used the chain rule $\nabla_{y}v=\nabla {\tilde{\phi }}_{s}^{-T}\nabla \tilde{v}$, the notation of d-by- d symmetric tensor

(54) $$E\left(M,\tilde{v}\right):=\frac{1}{2}\left(M\nabla \tilde{v}+\nabla {\tilde{v}}^{T}M^{T}\right)\;\mathrm{for}\;M\in R^{d\times d},$$

such that $E(I,\tilde{v})=\varepsilon (\tilde{v})$ according to equation (4), the Jacobian determinant

(55) $$J_{s}:=\det\left(\nabla \phi_{s}\right)\;\mathrm{in}\;\Omega_{t},\;\omega_{s}:=|\nabla {\tilde{\phi }}_{s}^{-T}n_{t}|J_{s}\;\mathrm{at}\;\Gamma_{t},$$

and the fact that $\phi_{s}$ is the identity transformation at $\Gamma_{N}$.

### Trait 3 (asymptotic expansion)

The asymptotic expansion of $\tilde{L}$ from equation (53) as $s\to 0^{+}$ holds

(56) $$\tilde{L}\left(s,\tilde{v},\tilde{q},\tilde{\mu };\Gamma_{t}\right)=L\left(\tilde{v},\tilde{q},\tilde{\mu };\Gamma_{t}\right)+s\frac{\partial }{\partial s}\tilde{L}\left(0,\tilde{v},\tilde{q},\tilde{\mu };\Gamma_{t}\right)+o\left(s\right).$$

The partial derivative $\partial \tilde{L}/\partial s$ in equation (56) is a continuous function of the first argument given by the explicit representation for τ∈[0,T−t)

(57) $$\begin{array}{c} \frac{\partial }{\partial s}\tilde{L}\left(\tau ,\tilde{v},\tilde{q},\tilde{\mu };\Gamma_{t}\right):=\int_{\Omega_{t}}\{d\mathrm{iv}\;\Lambda {|}_{t+\tau }\left(\frac{1}{2}A\varepsilon \left(\tilde{v}\right)+\tau^{0}-\alpha \tilde{q}I\right):\varepsilon \left(\tilde{v}\right)+\Lambda {|}_{t+\tau }^{T}\left(\frac{1}{2}\nabla A\varepsilon \left(\tilde{v}\right)+\nabla \tau^{0}\right):\varepsilon \left(\tilde{v}\right)\\ \;-\left(A\varepsilon \left(\tilde{v}\right)+\tau^{0}-\alpha \tilde{q}I\right):E\left(\nabla \Lambda {|}_{t+\tau }^{T},\tilde{v}\right)-d\mathrm{iv}\;\Lambda {|}_{t+\tau }\left[S\left(\frac{1}{2}\tilde{q}-p_{t-\delta }\right)-\alpha tr\;\varepsilon \left(u_{t-\delta }\right)\right]\tilde{q}\\ +\Lambda {|}_{t+\tau }^{T}\nabla \left(Sp_{t-\delta }+\alpha tr\;\varepsilon \left(u_{t-\delta }\right)\right)\tilde{q}-\delta \left[\kappa \left(\frac{1}{2}d\mathrm{iv}\;\Lambda {|}_{t+\tau }|\nabla \tilde{q}{|}^{2}-\nabla {\tilde{q}}^{T}\nabla \Lambda {|}_{t+\tau }\nabla \tilde{q}\right)+\frac{1}{2}\Lambda {|}_{t+\tau }^{T}\nabla \kappa |\nabla \tilde{q}{|}^{2}\right]\}dx\\ \;-\int_{\Gamma_{t}}\left(\left(div_{\Gamma_{t}}\Lambda {|}_{t+\tau }n_{t}^{T}+\Lambda {|}_{t+\tau }^{T}\nabla n_{t}^{T}\right)\left[\left[p_{re}\tilde{v}\right]\right]+\Lambda {|}_{t+\tau }^{T}\left[\left[\nabla p_{re}{\tilde{v}}^{T}\right]\right]n_{t}\right)dS_{x}\\ \;+{〈\tilde{\mu },\left(div_{\Gamma_{t}}\Lambda {|}_{t+\tau }n_{t}^{T}+\Lambda {|}_{t+\tau }^{T}\nabla n_{t}^{T}\right)\left[\left[\tilde{v}\right]\right]〉}_{\Gamma_{t}}, \end{array}$$

where the tangential divergence $div_{\Gamma_{t}}\Lambda :=d\mathrm{iv}\Lambda -n_{t}^{T}\Lambda n_{t}$ at $\Gamma_{t}$.

### Proof

As s→0, the following asymptotic expansion of the terms entering equations (53)–(55) takes place (see, e.g., Sokolowski and Zolesio [35], Chapter 2)

(58) $$\begin{array}{c} \tilde{A}=A+s\Lambda^{T}\nabla A+o\left(s\right),\;{\tilde{\tau }}^{0}=\tau^{0}+s\Lambda^{T}\nabla \tau^{0}+o\left(s\right),\;{\tilde{n}}_{t+s}=n_{t}+s\nabla n_{t}\Lambda +o\left(s\right),\;\tilde{\kappa }=\kappa +s\Lambda^{T}\nabla \kappa +o\left(s\right),\\ \;{\tilde{p}}_{\mathrm{re}}=p_{re}+s\Lambda^{T}\nabla p_{re}+o\left(s\right),\;{\tilde{p}}_{t-\delta }=p_{t-\delta }+s\Lambda^{T}\nabla p_{t-\delta }+o\left(s\right),\;J_{s}=1+sd\mathrm{iv}\Lambda +o\left(s\right),\\ \omega_{s}=1+s\;div_{\Gamma_{t}}\Lambda +o\left(s\right),\;\nabla {\tilde{\phi }}_{s}^{-T}=I-s\nabla \Lambda^{T}+o\left(s\right),\;E\left(\nabla {\tilde{\phi }}_{s}^{-T},\tilde{v}\right)=\varepsilon \left(\tilde{v}\right)-sE\left(\nabla \Lambda^{T},\tilde{v}\right)+o\left(s\right),\\ \;{\tilde{u}}_{t-\delta }=u_{t-\delta }+s\nabla u_{t-\delta }\Lambda +o\left(s\right),\;tr\;E\left(\nabla {\tilde{\phi }}_{s}^{-T},{\tilde{u}}_{t-\delta }\right)=tr\;\varepsilon \left(u_{t-\delta }\right)+s\Lambda^{T}\nabla \left[tr\;\varepsilon \left(u_{t-\delta }\right)\right]+o(s). \end{array}$$

Inserting representations (58) into the Lagrangian $\tilde{L}$ given by equation (53), we derive its expansions (56) in the first argument. Since $\Lambda {|}_{t+\tau }$ is a continuous function of the argument t+τ, then the partial derivative $\tau \mapsto \partial \tilde{L}/\partial s(\tau ,\cdot )$ in equation (57) is continuous and implies $\Lambda {|}_{t+\tau }=\Lambda$ at τ=0. This finishes the proof.

The last trait is rather involved and proven in Appendix 1.

### Trait 4 (strong convergence)

There exists a subsequence of saddle–points $\left({\tilde{u}}_{t+s_{k}},{\tilde{p}}_{t+s_{k}}-p_{re},{\tilde{\lambda }}_{t+s_{k}}\right)$ in equation (52) converging for $s_{k}\to 0$ as k→∞ to the saddle-point $\left(u_{t},p_{t}-p_{\mathrm{re}},\lambda_{t}\right)$ in equation (33)

(59) $$\left({\tilde{u}}_{t+s_{k}},{\tilde{p}}_{t+s_{k}},{\tilde{\lambda }}_{t+s_{k}}\right)\to \left(u_{t},p_{t},\lambda_{t}\right)\;\mathrm{strongly}\;\mathrm{in}\;H^{1}{\left(\Omega_{t}\right)}^{d}\times H^{1}\left(\Omega_{t}\right)\times H_{00}^{1/2}{\left(\Gamma_{t}\right)}^{★}.$$

Traits 1–4 satisfy all assumptions in Delfour and Zolésio [28] (Chapter 10, Theorem 5.1), thus provide the following theorem (see the detailed proof in González et al. [29]).

### Theorem 2 (shape differentiability of Lagrangian)

The shape derivative from equation (41) exists expressed by

(60) $$\begin{array}{c} \frac{\partial }{\partial t}L\left(u_{t},p_{t},\lambda_{t};\Gamma_{t}\right)=\underset{s_{k}\to 0^{+}}{\lim}\frac{1}{s_{k}}\left(\tilde{L}\left(s_{k},{\tilde{u}}_{t+s_{k}},{\tilde{p}}_{t+s_{k}},{\tilde{\lambda }}_{t+s_{k}};\Gamma_{t}\right)-L\left(u_{t},p_{t},\lambda_{t};\Gamma_{t}\right)\right)\\ \;=\frac{\partial }{\partial s}\tilde{L}\left(0,u_{t},p_{t},\lambda_{t};\Gamma_{t}\right), \end{array}$$

where $\left(u_{t},p_{t},\lambda_{t}\right)$ is the solution to the poroelastic problem with non-penetrating crack (28)–(31), and formula for the partial derivative $\partial /\partial s\tilde{L}$ is given in equation (57).

In the following, we specify our main result stated in Theorem 2 with respect to the so-called J-integrals well-known in brittle fracture for linear elastic bodies with cracks.

## 5. Representation of the energy release rate as J-integral

For the kinematic velocity Λ from equations (46) and (47), let there exists a d-dimensional set O⊂Ω with the $C^{1,1}$-smooth boundary ∂O and outward normal n such that outside it the solution to equations (28)–(31) is regular

(61) $$\left(u_{t},p_{t},\lambda_{t}\right)\in H^{2}{\left(\Omega_{t}\backslash O\right)}^{d}\times H^{2}\left(\Omega_{t}\backslash O\right)\times L^{2}\left(\Gamma_{t}\backslash O\right).$$

Typically, O surrounds crack-tip, crack-front, kinks, and other singular points, where singular solutions are locally admissible. Inside O, we assume the velocity constant, e.g., equal to one, such that

(62) ∇Λ≡0inO.

We denote for short $O_{t}:=O\backslash \bar{\Gamma_{t}}$. Based on properties (61) and (62), in the following, we will integrate by parts the expression in equation (57).

### Theorem 3 (J-integral)

Under assumptions (61) and (62), the shape derivative in equation (60) for the solution of the poroelastic problem with non-penetrating crack (28)–(31) admits equivalent representation by the following sum

(63) $$\frac{\partial }{\partial s}\tilde{L}\left(0,u_{t},p_{t},\lambda_{t};\Gamma_{t}\right)=J_{O_{t}}+J_{\partial O\backslash \Gamma_{t}}+J_{\Gamma_{t}\cap O}+J_{\Gamma_{t}\backslash O}+J_{\partial O\cap \Gamma_{t}},$$

where the integrals are

(64) $$J_{O_{t}}:=\int_{O_{t}}\left\{\Lambda^{T}\left(\frac{1}{2}\nabla A\varepsilon \left(u_{t}\right)+\nabla \tau^{0}\right):\varepsilon \left(u_{t}\right)+\Lambda^{T}\nabla \left(Sp_{t-\delta }+\alpha tr\;\varepsilon \left(u_{t-\delta }\right)\right)p_{t}-\frac{\delta }{2}\Lambda^{T}\nabla \kappa |\nabla p_{t}{|}^{2}\right\}dx,$$

(65) $$\begin{array}{c} J_{\partial O\backslash \Gamma_{t}}:=\int_{\partial O\backslash \Gamma_{t}}\{\left(\Lambda^{T}n\right)\left[\left(\frac{1}{2}A\varepsilon \left(u_{t}\right)+\tau^{0}-\alpha p_{t}I\right):\varepsilon \left(u_{t}\right)-\left(S\left(\frac{1}{2}p_{t}-p_{t-\delta }\right)-\alpha tr\;\varepsilon \left(u_{t-\delta }\right)\right)p_{t}\right]\\ \;-\Lambda^{T}\nabla u_{t}^{T}\tau_{t}n-\delta \kappa \left(\frac{1}{2}\left(\Lambda^{T}n\right)|\nabla p_{t}{|}^{2}-\left(\Lambda^{T}\nabla p_{t}\right)\left(n^{T}\nabla p_{t}\right)\right)\}dS_{x}, \end{array}$$

(66) $$\begin{array}{c} J_{\Gamma_{t}\cap O}:={〈\lambda_{t},\Lambda^{T}\nabla n_{t}^{T}\left[\left[u_{t}\right]\right]〉}_{\Gamma_{t}\cap O}-\int_{\Gamma_{t}\cap O}\Lambda^{T}\left(\nabla n_{t}^{T}\left[\left[p_{re}u_{t}\right]\right]+\left[\left[\nabla p_{re}u_{t}^{T}\right]\right]n_{t}\right)dS_{x},\\ \mathrm{using}\;\mathrm{the}\;\mathrm{notation}\;{〈\lambda_{t},\Lambda^{T}\nabla n_{t}^{T}\left[\left[u_{t}\right]\right]〉}_{\Gamma_{t}\cap O}:={〈\lambda_{t},\Lambda^{T}\nabla n_{t}^{T}\left[\left[u_{t}\right]\right]〉}_{\Gamma_{t}}-\int_{\Gamma_{t}\backslash O}\lambda_{t}\Lambda^{T}\nabla n_{t}^{T}\left[\left[u_{t}\right]\right]dS_{x}, \end{array}$$

(67) $$J_{\Gamma_{t}\backslash O}:=-\delta \int_{\Gamma_{t}\backslash O}\Lambda^{T}\kappa \left[\left[\nabla p_{t}\nabla p_{t}^{T}\right]\right]n_{t}dS_{x},$$

(68) $$J_{\partial O\cap \Gamma_{t}}:=-\left\{ \begin{array}{cc} \left(\Lambda^{T}t_{t}\right)n_{t}^{T}{\left[\left[p_{re}u_{t}\right]\right]}_{\partial O\cap \Gamma_{t}} & \mathrm{in}\;2D,\\ \int_{\partial O\cap \Gamma_{t}}\left(\Lambda^{T}b_{t}\right)n_{t}^{T}\left[\left[p_{re}u_{t}\right]\right]dL_{x} & \mathrm{in}\;3D, \end{array}\right.$$

and the effective stress $\tau_{t}=A\varepsilon (u_{t})+\tau^{0}-\alpha p_{t}I$. In equation (68) $t_{t}$ is a tangential vector at $\partial \Gamma_{t}$ positive oriented to $n_{t}$ in 2D, and $b_{t}=t_{t}\times n_{t}$ is a binomial vector within the moving frame at $\partial \Gamma_{t}$ in 3D.

### Proof

We rearrange the terms in formula (57) on the solution $\left(u_{t},p_{t},\lambda_{t}\right)$ in the sum

(69) $$\frac{\partial }{\partial s}\tilde{L}\left(0,u_{t},p_{t},\lambda_{t};\Gamma_{t}\right)=\sum_{k=0}^{6}I_{k}.$$

The terms not including ∇Λ are gathered in

$$\begin{array}{c} I_{1}:=\int_{\Omega_{t}}\left\{\Lambda^{T}\left(\frac{1}{2}\nabla A\varepsilon \left(u_{t}\right)+\nabla \tau^{0}\right):\varepsilon \left(u_{t}\right)+\Lambda^{T}\nabla \left(Sp_{t-\delta }+\alpha tr\;\varepsilon \left(u_{t-\delta }\right)\right)p_{t}-\frac{\delta }{2}\Lambda^{T}\nabla \kappa |\nabla p_{t}{|}^{2}\right\}dx\\ \;-\int_{\Gamma_{t}}\Lambda^{T}\left(\nabla n_{t}^{T}\left[\left[p_{re}u_{t}\right]\right]+\left[\left[\nabla p_{re}u_{t}^{T}\right]\right]n_{t}\right)dS_{x}+{〈\lambda_{t},\Lambda^{T}\nabla n_{t}^{T}\left[\left[u_{t}\right]\right]〉}_{\Gamma_{t}}. \end{array}$$

Since the assumptions of regularity (61) and (62), the complementarity conditions in equation (25) hold pointwise at $\Gamma_{t}\backslash O$, therefore

$$I_{0}:={〈\lambda_{t},div_{\Gamma_{t}}\Lambda n_{t}^{T}\left[\left[u_{t}\right]\right]〉}_{\Gamma_{t}}=\int_{\Gamma_{t}\backslash O}\left(div_{\Gamma_{t}}\Lambda \right)\lambda_{t}n_{t}^{T}\left[\left[u_{t}\right]\right]dS_{x}=0.$$

In $I_{2},\ldots ,I_{6}$, we integrating by parts with respect to ∇Λ. Using $\tau_{t}=A\varepsilon (u_{t})+\tau^{0}-\alpha p_{t}I$, we calculate

$$\begin{array}{c} I_{2}:=\int_{\Omega_{t}\backslash O}d\mathrm{iv}\;\Lambda \left(\frac{1}{2}A\varepsilon \left(u_{t}\right)+\tau^{0}-\alpha p_{t}I\right):\varepsilon \left(u_{t}\right)dx=\int_{\partial O\backslash \Gamma_{t}}\left\{\left(\Lambda^{T}n\right)\left(\frac{1}{2}A\varepsilon \left(u_{t}\right)+\tau^{0}-\alpha p_{t}I\right):\varepsilon \left(u_{t}\right)\right\}dS_{x}\\ \;+\int_{\Omega_{t}\backslash O}\left\{-\Lambda^{T}\left(\frac{1}{2}\nabla A\varepsilon \left(u_{t}\right)+\nabla \tau^{0}\right):\varepsilon \left(u_{t}\right)+\alpha \Lambda^{T}\nabla p_{t}tr\varepsilon \left(u_{t}\right)-\Lambda^{T}\nabla \varepsilon \left(u_{t}\right):\tau_{t}\right\}dx, \end{array}$$

where the last term $\Lambda^{T}\nabla \varepsilon (u_{t}):\tau_{t}$ will be shortened when adding

$$\begin{array}{c} I_{3}:=-\int_{\Omega_{t}\backslash O}\tau_{t}:E\left(\nabla \Lambda^{T},u_{t}\right)dx=\int_{\Omega_{t}\backslash O}\left\{\sum_{i=1}^{d}\Lambda^{T}\nabla {\left(u_{t}\right)}_{i}{\left(d\mathrm{iv}\;\tau_{t}\right)}_{i}+\Lambda^{T}\nabla \varepsilon \left(u_{t}\right):\tau_{t}\right\}dx\\ \;-\int_{\partial O\backslash \Gamma_{t}}\Lambda^{T}\nabla u_{t}^{T}\tau_{t}ndS_{x}+\int_{\Gamma_{t}\backslash O}\Lambda^{T}\left[\left[\nabla u_{t}^{T}\tau_{t}\right]\right]n_{t}dS_{x}, \end{array}$$

and $d\mathrm{iv}\;\tau_{t}=0$ due to the equilibrium equation (3), then

$$\begin{array}{c} I_{4}:=-\int_{\Omega_{t}\backslash O}d\mathrm{iv}\;\Lambda \left(S\left(\frac{1}{2}p_{t}-p_{t-\delta }\right)-\alpha tr\;\varepsilon \left(u_{t-\delta }\right)\right)p_{t}dx=\int_{\Omega_{t}\backslash O}\{\Lambda^{T}\nabla p_{t}\left(S\left(p_{t}-p_{t-\delta }\right)-\alpha tr\;\varepsilon \left(u_{t-\delta }\right)\right)\\ \;-\Lambda^{T}\nabla \left(Sp_{t-\delta }+\alpha tr\;\varepsilon \left(u_{t-\delta }\right)\right)p_{t}\}dx-\int_{\partial O\backslash \Gamma_{t}}\left(\Lambda^{T}n\right)\left(S\left(\frac{1}{2}p_{t}-p_{t-\delta }\right)-\alpha tr\;\varepsilon \left(u_{t-\delta }\right)\right)p_{t}dS_{x}, \end{array}$$

which due to equation (10) constitutes the zeroth term

$$\Lambda^{T}\nabla p_{t}\left[S\left(p_{t}-p_{t-\delta }\right)+\alpha tr\;\varepsilon \left(u_{t}-u_{t-\delta }\right)-\delta d\mathrm{iv}\left(\kappa \nabla p_{t}\right)\right]=0,$$

together with $I_{2}$ and

$$\begin{array}{c} I_{5}:=-\int_{\Omega_{t}\backslash O}\delta \kappa \left(\frac{1}{2}d\mathrm{iv}\;\Lambda |\nabla p_{t}{|}^{2}-\nabla p_{t}^{T}\nabla \Lambda \nabla p_{t}\right)dx=\int_{\Omega_{t}\backslash O}\delta \left(\frac{1}{2}\Lambda^{T}\nabla \kappa |\nabla p_{t}{|}^{2}-\Lambda^{T}\nabla p_{t}d\mathrm{iv}\left(\kappa \nabla p_{t}\right)\right)dx\\ \;-\int_{\partial O\backslash \Gamma_{t}}\delta \kappa \left(\frac{1}{2}\left(\Lambda^{T}n\right)|\nabla p_{t}{|}^{2}-\left(\Lambda^{T}\nabla p_{t}\right)\left(n^{T}\nabla p_{t}\right)\right)dS_{x}-\int_{\Gamma_{t}\backslash O}\delta \kappa \Lambda^{T}\left[\left[\nabla p_{t}\nabla p_{t}^{T}\right]\right]n_{t}dS_{x}. \end{array}$$

For $I_{6}$, differentiating along the crack gets

$$\begin{array}{c} I_{6}:=-\int_{\Gamma_{t}\backslash O}div_{\Gamma_{t}}\Lambda n_{t}^{T}\left[\left[p_{\mathrm{re}}u_{t}\right]\right]dS_{x}=\int_{\Gamma_{t}\backslash O}\Lambda^{T}\left(\left[\left[\nabla p_{\mathrm{re}}u_{t}^{T}\right]\right]n_{t}+\nabla n_{t}^{T}\left[\left[p_{\mathrm{re}}u_{t}\right]\right]+\left[\left[\nabla u_{t}^{T}p_{\mathrm{re}}\right]\right]n_{t}\right)dS_{x}\\ \;-\left(\Lambda^{T}t_{t}\right)n_{t}^{T}{\left[\left[p_{\mathrm{re}}u_{t}\right]\right]}_{\partial O\cap \Gamma_{t}}\mathrm{in}\;2D,\;\mathrm{or}\;-\int_{\partial O\cap \Gamma_{t}}\left(\Lambda^{T}b_{t}\right)n_{t}^{T}\left[\left[p_{\mathrm{re}}u_{t}\right]\right]dL_{x}\;\mathrm{in}\;3D, \end{array}$$

where in 2D a tangential vector $t_{t}$ at $\partial \Gamma_{t}$ is positive oriented to $n_{t}$, and in 3D a binomial vector $b_{t}=t_{t}\times n_{t}$ builds the moving frame at $\partial \Gamma_{t}$.

The integrals from $I_{3}$ and $I_{6}$ over $\Gamma_{t}\backslash O$, where the stress is determined pointwisely, can be combined using the boundary conditions (21), (23), definition of $\lambda_{t}$ in equation (29), and the following calculation

(70) $$\begin{array}{c} \int_{\Gamma_{t}\backslash O}\Lambda^{T}\left(\lambda_{t}\nabla n_{t}^{T}\left[\left[u_{t}\right]\right]+\left[\left[\nabla u_{t}^{T}\tau_{t}\right]\right]n_{t}+\left[\left[\nabla u_{t}^{T}p_{re}\right]\right]n_{t}\right)dS_{x}=\int_{\Gamma_{t}\backslash O}\Lambda^{T}\left(\lambda_{t}\nabla n_{t}^{T}\left[\left[u_{t}\right]\right]+\left[\left[\nabla u_{t}^{T}\right]\right]\lambda_{t}n_{t}\right)dS_{x}\\ \;=\int_{\Gamma_{t}\backslash O}\Lambda^{T}\lambda_{t}\nabla \left(n_{t}^{T}\left[\left[u_{t}\right]\right]\right)dS_{x}=\int_{\Gamma_{t}\backslash O}\Lambda^{T}\lambda_{t}\nabla_{\Gamma_{t}}\left(n_{t}^{T}\left[\left[u_{t}\right]\right]\right)dS_{x}=0, \end{array}$$

by decomposing the vectors into normal and tangential components and using $\Lambda^{T}n_{t}=0$ at the crack $\Gamma_{t}$. The pointwise product in equation (70) is zero due to the complementarity conditions (25) after application of the tangential differentiation $\nabla_{\Gamma_{t}}:=\nabla -n_{t}(n_{t}^{T}\nabla )$ to $n_{t}^{T}[[u_{t}]]=0$. Finally, collecting the integral terms $I_{1}$– $I_{6}$ in equation (69) together with equation (70) provides the expressions (63)–(68).

We present several concluding remarks as follows:

- With the help of tangential gradient and $\Lambda^{T}n_{t}=0$ at $\Gamma_{t}$, formula (66) can be expressed equivalently

$$J_{\Gamma_{t}\cap O}=〈\lambda_{t},\Lambda^{T}\nabla_{\Gamma_{t}}n_{t}^{T}\left[\left[u_{t}\right]\right]{〉}_{\Gamma_{t}\cap O}-\int_{\Gamma_{t}\cap O}\Lambda^{T}\left(\nabla_{\Gamma_{t}}n_{t}^{T}\left[\left[p_{re}u_{t}\right]\right]+\left[\left[\nabla_{\Gamma_{t}}p_{re}u_{t}^{T}\right]\right]n_{t}\right)dS_{x}.$$

- Applying the coordinate transformation $\phi_{s}$, using expansion of $\omega_{s}$ in equation (58), and differentiating along the crack, we calculate the limit in equation (42)

$$G_{t}=2\gamma \underset{s\to 0^{+}}{\lim}\frac{1}{s}\int_{\Gamma_{t}}\left(\omega_{s}-1\right)dS_{x}=2\gamma \left\{ \begin{array}{cc} \Lambda^{T}t_{t}{|}_{\partial \Gamma_{t}} & \mathrm{in}\;2D,\\ \int_{\partial \Gamma_{t}}\Lambda^{T}b_{t}dL_{x} & \mathrm{in}\;3D. \end{array}\right.$$

- It is worth noting that $J_{\partial O\backslash \Gamma_{t}}$ from equation (66) is related to J-integrals known for linear elastic bodies. We clarify the relation in the following corollary.

### Corollary 1 (non-penetrating crack in linear elastic body under fluid-driven fracture)

If the factors S=α=κ=0, then the Lagrangian L in equation (32) implies the strain energy

(71) $$L\left(u,p,\lambda ;\Gamma_{t}\right)=\int_{\Omega_{t}}\left(\frac{1}{2}A\varepsilon \left(u\right)+\tau^{0}\right):\varepsilon \left(u\right)dx-\int_{\Gamma_{N}}g^{T}udS_{x}-\int_{\Gamma_{t}}n_{t}^{T}\left[\left[p_{re}u\right]\right]dS_{x}+{〈\lambda ,n_{t}^{T}\left[\left[u\right]\right]〉}_{\Gamma_{t}}.$$

The strain energy release rate is given according to equation (57) by

$$\begin{array}{c} \frac{\partial }{\partial s}\tilde{L}\left(0,u_{t},p_{t},\lambda_{t};\Gamma_{t}\right)=\int_{\Omega_{t}}\{d\mathrm{iv}\;\Lambda \left(\frac{1}{2}A\varepsilon \left(u_{t}\right)+\tau^{0}\right):\varepsilon \left(u_{t}\right)\\ \;+\Lambda^{T}\left(\frac{1}{2}\nabla A\varepsilon \left(u_{t}\right)+\nabla \tau^{0}\right):\varepsilon \left(u_{t}\right)-\left(A\varepsilon \left(u_{t}\right)+\tau^{0}\right):E\left(\nabla \Lambda^{T},u_{t}\right)\}dx\\ -\int_{\Gamma_{t}}\left(\left(div_{\Gamma_{t}}\Lambda n_{t}^{T}+\Lambda^{T}\nabla n_{t}^{T}\right)\left[\left[p_{re}u_{t}\right]\right]+\Lambda^{T}\left[\left[\nabla p_{re}u_{t}^{T}\right]\right]n_{t}\right)dS_{x}+{〈\lambda_{t},\left(div_{\Gamma_{t}}\Lambda n_{t}^{T}+\Lambda^{T}\nabla n_{t}^{T}\right)\left[\left[u_{t}\right]\right]〉}_{\Gamma_{t}}. \end{array}$$

Let the elasticity coefficients A and prestress $\tau^{0}$ be constant. Then, under assumptions (61) and (62), the shape derivative is expressed equivalently by the integrals from equations (63)–(68)

(72) $$\begin{array}{c} \frac{\partial }{\partial s}\tilde{L}\left(0,u_{t},p_{t},\lambda_{t};\Gamma_{t}\right)=\int_{\partial O\backslash \Gamma_{t}}\left\{\left(\Lambda^{T}n\right)\left(\frac{1}{2}A\varepsilon \left(u_{t}\right)+\tau^{0}\right):\varepsilon \left(u_{t}\right)-\Lambda^{T}\nabla u_{t}^{T}\tau_{t}n\right\}dS_{x}\\ \;+J_{\Gamma_{t}\cap O}+J_{\partial O\cap \Gamma_{t}}. \end{array}$$

If $p_{re}$ is constant and the crack $\Gamma_{t}$ is plane in O, then $\nabla p_{re}=\nabla n_{t}=0$ at $\Gamma_{t}\cap O$ such that $J_{\Gamma_{t}\cap O}=0$, and the strain energy release rate in equation (72) implies the path-independent sum

(73) $$\frac{\partial }{\partial s}\tilde{L}\left(0,u_{t},p_{t},\lambda_{t};\Gamma_{t}\right)=\int_{\partial O\backslash \Gamma_{t}}\left\{\left(\Lambda^{T}n\right)\left(\frac{1}{2}A\varepsilon \left(u_{t}\right)+\tau^{0}\right):\varepsilon \left(u_{t}\right)-\Lambda^{T}\nabla u_{t}^{T}\tau_{t}n\right\}dS_{x}+J_{\partial O\cap \Gamma_{t}}.$$

- We finish the paper by emphasizing that formulas of the shape derivative obtained in the paper are of practical use to predict when fluid-driven fractures will begin to grow.

## Appendix 1

### Proof of the strong convergence in Trait 4

We split the proof in five subsequent steps.

#### Uniform estimate of $\left({\tilde{u}}_{t+s},{\tilde{p}}_{t+s}\right)$

Applying Theorem 1 to the s-dependent problem (52), its saddle-point $\left({\tilde{u}}_{t+s},{\tilde{p}}_{t+s}-p_{re},{\tilde{\lambda }}_{t+s})\in K(\Omega_{t}\right)$ satisfies the necessary and sufficient optimality conditions like (38)–(40)

(74)
$$\begin{array}{c} \int_{\Omega_{t}}\left(\tilde{A}E\left(\nabla \phi_{s}^{-T},{\tilde{u}}_{t+s}\right)+{\tilde{\tau }}^{0}-\alpha {\tilde{p}}_{t+s}I\right):E\left(\nabla \phi_{s}^{-T},\tilde{v}\right)J_{s}dx\\ \;-\int_{\Gamma_{N}}g^{T}\tilde{v}dS_{x}-\int_{\Gamma_{t}}{\tilde{n}}_{t+s}^{T}\left[\left[{\tilde{p}}_{r}\tilde{v}\right]\right]\omega_{s}dS_{x}+{〈\tilde{\mu },{\tilde{n}}_{t+s}^{T}\left[\left[\tilde{v}\right]\right]\omega_{s}〉}_{\Gamma_{t}}=0, \end{array}$$

(75)
$$\int_{\Omega_{t}}\left(\left[S\left({\tilde{p}}_{t+s}-{\tilde{p}}_{t-\delta }\right)+\alpha tr\;E\left(\nabla {\tilde{\phi }}_{s}^{-T},{\tilde{u}}_{t+s}-{\tilde{u}}_{t-\delta }\right)\right]\tilde{q}+\delta \tilde{\kappa }\nabla {\tilde{p}}_{t+s}^{T}\nabla {\tilde{\phi }}_{s}^{-1}\nabla {\tilde{\phi }}_{s}^{-T}\nabla \tilde{q}\right)J_{s}dx=0,$$

(76)
$${\tilde{n}}_{t+s}^{T}\left[\left[{\tilde{u}}_{t+s}\right]\right]\geq 0,\;{〈{\tilde{\lambda }}_{t+s},\xi -{\tilde{n}}_{t+s}^{T}\left[\left[{\tilde{u}}_{t+s}\right]\right]\omega_{s}〉}_{\Gamma_{t}}\leq 0\;\mathrm{for}\;\mathrm{all}\;\xi \in H_{00}^{1/2}\left(\Gamma_{t}\right)\mathrm{such}\;\mathrm{that}\;\xi \geq 0,$$

for all test functions $\left(\tilde{v},\tilde{q})\in H^{1}(\Omega_{t}{)}^{d}\times H_{0}^{1}(\Omega_{t}\right)$ such that $\tilde{v}=0$ on $\Gamma_{D}$. With the help of asymptotic expansion (56) and (57) in Trait 3 and applying Taylor’s theorem, we can decompose equations (74)–(76) as $s\to 0^{+}$ akin to the reference problem (28)–(31)

(77)
$$\begin{array}{c} \int_{\Omega_{t}}\left(A\varepsilon \left({\tilde{u}}_{t+s}\right)+\tau^{0}-\alpha {\tilde{p}}_{t+s}I\right):\varepsilon \left(\tilde{v}\right)dx-\int_{\Gamma_{N}}g^{T}\tilde{v}dS_{x}-\int_{\Gamma_{t}}n_{t}^{T}\left[\left[p_{re}\tilde{v}\right]\right]dS_{x}+{〈{\tilde{\lambda }}_{t+s},{\tilde{n}}_{t+s}^{T}\left[\left[\tilde{v}\right]\right]\omega_{s}〉}_{\Gamma_{t}}\\ \;=sB_{u}\left(s,\left({\tilde{u}}_{t+s},{\tilde{p}}_{t+s}\right),\tilde{v}\right), \end{array}$$

(78)
$$\int_{\Omega_{t}}\left(\left[S\left({\tilde{p}}_{t+s}-p_{t-\delta }\right)+\alpha tr\;\varepsilon \left({\tilde{u}}_{t+s}-u_{t-\delta }\right)\right]\tilde{q}+\delta \kappa \nabla {\tilde{p}}_{t+s}^{T}\nabla \tilde{q}\right)dx=sB_{p}\left(s,\left({\tilde{u}}_{t+s},{\tilde{p}}_{t+s}\right),\tilde{q}\right),$$

(79)
$${〈{\tilde{\lambda }}_{t+s},{\tilde{n}}_{t+s}^{T}\left[\left[\tilde{v}\right]\right]\omega_{s}〉}_{\Gamma_{t}}={〈{\tilde{\lambda }}_{t+s},n_{t}^{T}\left[\left[\tilde{v}\right]\right]〉}_{\Gamma_{t}}+sB_{\lambda }\left(s,{\tilde{\lambda }}_{t+s},\tilde{v}\right),$$

where the remainders in the form of Lagrange build the bilinear forms

(80)
$$\begin{array}{c} B_{u}\left(s,\left({\tilde{u}}_{t+s},{\tilde{p}}_{t+s}\right),\tilde{v}\right):=-\int_{\Omega_{t}}\{\Lambda {|}_{t+s\theta_{u}}^{T}\left(\nabla A\varepsilon \left({\tilde{u}}_{t+s}\right)+\nabla \tau^{0}\right):\varepsilon \left(\tilde{v}\right)\\ \;\;+\left(A\varepsilon \left({\tilde{u}}_{t+s}\right)+\tau^{0}-\alpha {\tilde{p}}_{t+s}I\right):\left(d\mathrm{iv}\Lambda {|}_{t+s\theta_{u}}\varepsilon \left(\tilde{v}\right)-E\left(\nabla \Lambda {|}_{t+s\theta_{u}}^{T},\tilde{v}\right)\right)\}dx\\ \;+\int_{\Gamma_{t}}\left(\left(div_{\Gamma_{t}}\Lambda {|}_{t+s\theta_{u}}n_{t}^{T}+\Lambda {|}_{t+s\theta_{u}}^{T}\nabla n_{t}^{T}\right)\left[\left[p_{re}\tilde{v}\right]\right]+\Lambda {|}_{t+s\theta_{u}}^{T}\left[\left[\nabla p_{re}{\tilde{v}}^{T}\right]\right]n_{t}\right)dS_{x}, \end{array}$$

(81)
$$\begin{array}{c} B_{p}\left(s,\left({\tilde{u}}_{t+s},{\tilde{p}}_{t+s}\right),\tilde{q}\right):=-\int_{\Omega_{t}}\{d\mathrm{iv}\Lambda {|}_{t+s\theta_{p}}\left[S\left({\tilde{p}}_{t+s}-p_{t-\delta }\right)+\alpha tr\;\varepsilon \left({\tilde{u}}_{t+s}-u_{t-\delta }\right)\right]\tilde{q}\\ \;+\Lambda {|}_{t+s\theta_{p}}^{T}\nabla \left[Sp_{t-\delta }+\alpha tr\;\varepsilon \left(u_{t-\delta }\right)\right]\tilde{q}-\delta \nabla {\tilde{p}}_{t+s}^{T}\left(\kappa d\mathrm{iv}\Lambda {|}_{t+s\theta_{p}}I-\left(\kappa I-\nabla \kappa^{T}\right)\Lambda {|}_{t+s\theta_{p}}\right)\nabla \tilde{q}\}dx, \end{array}$$

(82)
$$B_{\lambda }\left(s,{\tilde{\lambda }}_{t+s},\tilde{v}\right):={〈{\tilde{\lambda }}_{t+s},\left(div_{\Gamma_{t}}\Lambda {|}_{t+s\theta_{\lambda }}n_{t}^{T}+\Lambda {|}_{t+s\theta_{\lambda }}^{T}\nabla n_{t}^{T}\right)\left[\left[\tilde{v}\right]\right]〉}_{\Gamma_{t}},$$

which are bounded for the parameters $\theta_{u},\theta_{p},\theta_{\lambda }\in [0,1]$.

We test the variational equation (77) with $\tilde{v}={\tilde{u}}_{t+s}$ and use $〈{\tilde{\lambda }}_{t+s},{\tilde{n}}_{t+s}^{T}[[{\tilde{u}}_{t+s}]]\omega_{s}{〉}_{\Gamma_{t}}=0$ from equation (76)

(83)
$$\begin{array}{c} \int_{\Omega_{t}}\left(A\varepsilon \left({\tilde{u}}_{t+s}\right)+\tau^{0}-\alpha {\tilde{p}}_{t+s}I\right):\varepsilon \left({\tilde{u}}_{t+s}\right)dx=\int_{\Gamma_{N}}g^{T}{\tilde{u}}_{t+s}dS_{x}+\int_{\Gamma_{t}}n_{t}^{T}\left[\left[p_{re}{\tilde{u}}_{t+s}\right]\right]dS_{x}\\ \;+sB_{u}\left(s,\left({\tilde{u}}_{t+s},{\tilde{p}}_{t+s}\right),{\tilde{u}}_{t+s}\right), \end{array}$$

then insert $\tilde{q}={\tilde{p}}_{t+s}-p_{re}$ into equation (78) such that

(84)
$$\begin{array}{c} \int_{\Omega_{t}}\left(\left[S\left({\tilde{p}}_{t+s}-p_{t-\delta }\right)+\alpha tr\;\varepsilon \left({\tilde{u}}_{t+s}-u_{t-\delta }\right)\right]{\tilde{p}}_{t+s}+\delta \kappa |\nabla {\tilde{p}}_{t+s}{|}^{2}\right)dx=\int_{\Omega_{t}}(S\left({\tilde{p}}_{t+s}-p_{t-\delta }\right)\\ \;+\alpha tr\;\varepsilon \left({\tilde{u}}_{t+s}-u_{t-\delta }\right)p_{re}+\delta \kappa \nabla {\tilde{p}}_{t+s}^{T}\nabla p_{re})dx+sB_{p}\left(s,\left({\tilde{u}}_{t+s},{\tilde{p}}_{t+s}\right),{\tilde{p}}_{t+s}-p_{re}\right). \end{array}$$

After summation of equations (83) and (84), the term $\alpha {\tilde{p}}_{t+s}I:\varepsilon ({\tilde{u}}_{t+s})=\alpha tr\;\varepsilon ({\tilde{u}}_{t+s}){\tilde{p}}_{t+s}$ is shortened and

(85)
$$\begin{array}{c} \;\int_{\Omega_{t}}\left(A\varepsilon \left({\tilde{u}}_{t+s}\right):\varepsilon \left({\tilde{u}}_{t+s}\right)+S{\tilde{p}}_{t+s}^{2}+\delta \kappa |\nabla {\tilde{p}}_{t+s}{|}^{2}\right)dx=\int_{\Omega_{t}}(-\tau^{0}:\varepsilon \left({\tilde{u}}_{t+s}\right)+\left[Sp_{t-\delta }+\alpha tr\;\varepsilon \left(u_{t-\delta }\right)\right]{\tilde{p}}_{t+s}\\ +\left[S\left({\tilde{p}}_{t+s}-p_{t-\delta }\right)+\alpha tr\;\varepsilon \left({\tilde{u}}_{t+s}-u_{t-\delta }\right)\right]p_{re}+\delta \kappa \nabla {\tilde{p}}_{t+s}^{T}\nabla p_{re})dx+\int_{\Gamma_{N}}g^{T}{\tilde{u}}_{t+s}dS_{x}+\int_{\Gamma_{t}}n_{t}^{T}\left[\left[p_{re}{\tilde{u}}_{t+s}\right]\right]dS_{x}\\ \;+sB_{u}\left(s,\left({\tilde{u}}_{t+s},{\tilde{p}}_{t+s}\right),{\tilde{u}}_{t+s}\right)+sB_{p}\left(s,\left({\tilde{u}}_{t+s},{\tilde{p}}_{t+s}\right),{\tilde{p}}_{t+s}-p_{re}\right). \end{array}$$

Applying Young’s inequality, for elliptic A from equation (16) and positive κ from equation (17), the uniform estimate holds

(86)
$$∥{\tilde{u}}_{t+s}{∥}_{H^{1}(\Omega_{t})}^{2}+∥{\tilde{p}}_{t+s}{∥}_{H^{1}(\Omega_{t})}^{2}\leq C_{1},\;C_{1}>0,$$

for $s\leq s_{0}$ with sufficiently small $s_{0}>0$.

#### Uniform estimate of ${\tilde{\lambda }}_{t+s}$

From equations (77) and (79), we express the duality

(87)
$$\begin{array}{c} {〈{\tilde{\lambda }}_{t+s},n_{t}^{T}\left[\left[\tilde{v}\right]\right]〉}_{\Gamma_{t}}=-\int_{\Omega_{t}}\left(A\varepsilon \left({\tilde{u}}_{t+s}\right)+\tau^{0}-\alpha {\tilde{p}}_{t+s}I\right):\varepsilon \left(\tilde{v}\right)dx+\int_{\Gamma_{N}}g^{T}\tilde{v}dS_{x}+\int_{\Gamma_{t}}n_{t}^{T}\left[\left[p_{re}\tilde{v}\right]\right]dS_{x}\\ \;+sB_{u}\left(s,\left({\tilde{u}}_{t+s},{\tilde{p}}_{t+s}\right),\tilde{v}\right)-sB_{\lambda }\left(s,{\tilde{\lambda }}_{t+s},\tilde{v}\right). \end{array}$$

Dividing the equality (87) by the norm of $n_{t}^{T}[[\tilde{v}]]$, taking supremum over $\tilde{v}$, using the Cauchy–Schwartz inequality and the bound (86), by the surjectivity of the trace operator we estimate the dual norm

(88)
$$∥{\tilde{\lambda }}_{t+s}{∥}_{H_{00}^{1/2}{\left(\Gamma_{t}\right)}^{★}}=\underset{\tilde{v}\in H^{1}{\left(\Omega_{t}\right)}^{d}}{\sup}\frac{\left|{〈{\tilde{\lambda }}_{t+s},n_{t}^{T}\left[\left[\tilde{v}\right]\right]〉}_{\Gamma_{t}}\right|}{∥n_{t}^{T}\left[\left[\tilde{v}\right]\right]{∥}_{H_{00}^{1/2}(\Gamma_{t})}}\leq C_{2},\;C_{2}>0.$$

for $s\leq s_{0}$ and sufficiently small $s_{0}>0$.

#### Weak convergence

By the virtue of uniform estimates (86) and (88), there exists a subsequence $s_{k}\to 0^{+}$ as k→∞ and an accumulation point such that $\left(u_{t},p_{t}-p_{re},\lambda_{t})\in K(\Omega_{t}\right)$ and

(89)
$$\left({\tilde{u}}_{t+s_{k}},{\tilde{p}}_{t+s_{k}},{\tilde{\lambda }}_{t+s_{k}}\right)⇀\left(u_{t},p_{t},\lambda_{t}\right)\;\mathrm{weakly}\;\mathrm{in}\;H^{1}{\left(\Omega_{t}\right)}^{d}\times H^{1}\left(\Omega_{t}\right)\times H_{00}^{1/2}{\left(\Gamma_{t}\right)}^{★}.$$

By the compactness of embedding of the boundary traces, it follows that

(90)
$${\tilde{u}}_{t+s_{k}}\to u_{t}\;\mathrm{strongly}\;\mathrm{in}\;L^{\;2}\left(\Gamma_{N}\cup \Gamma_{t}^{+}\cup \Gamma_{t}^{-}\right)\mathrm{as}\;s_{k}\to 0.$$

Taking the limit in the $s_{k}$-dependent problem (74)–(76) as $s_{k}\to 0$ due to the expansions (77)–(79) and the convergences (89) and (90), the limit $\left(u_{t},p_{t},\lambda_{t}\right)$ solves the reference variational problem (28)–(31).

#### Strong convergence of $\left({\tilde{u}}_{t+s_{k}},{\tilde{p}}_{t+s_{k}}\right)$

Testing the reference equations (28) with $v=u_{t}$, where $〈\lambda_{t},n_{t}^{T}[[u_{t}]]{〉}_{\Gamma_{t}}=0$, and inserting $q=p_{t}-p_{re}$ into equation (31), after summation the term $\alpha p_{t}I:\varepsilon (u_{t})=\alpha tr\;\varepsilon (u_{t})p_{t}$ is shortened, and similarly to equation (85), we obtain

(91)
$$\begin{array}{c} \int_{\Omega_{t}}\left(A\varepsilon \left(u_{t}\right):\varepsilon \left(u_{t}\right)+{\mathrm{Sp}}_{t}^{2}+\delta \kappa |\nabla p_{t}{|}^{2}\right)dx=\int_{\Omega_{t}}(-\tau^{0}:\varepsilon \left(u_{t}\right)+\left[Sp_{t-\delta }+\alpha tr\;\varepsilon \left(u_{t-\delta }\right)\right]p_{t}\\ +\left[S\left(p_{t}-p_{t-\delta }\right)+\alpha tr\;\varepsilon \left(u_{t}-u_{t-\delta }\right)\right]p_{re}+\delta \kappa \nabla p_{t}^{T}\nabla p_{re})dx+\int_{\Gamma_{N}}g^{T}u_{t}dS_{x}+\int_{\Gamma_{t}}n_{t}^{T}\left[\left[p_{re}u_{t}\right]\right]dS_{x}. \end{array}$$

We subtract equation (91) from equation (85) and rearrange the terms as follows

(92)
$$\begin{array}{c} \int_{\Omega_{t}}\left(A\varepsilon \left({\tilde{u}}_{t+s}-u_{t}\right):\varepsilon \left({\tilde{u}}_{t+s}-u_{t}\right)+S{\left({\tilde{p}}_{t+s}-p_{t}\right)}^{2}+\delta \kappa |\nabla \left({\tilde{p}}_{t+s}-p_{t}\right){|}^{2}\right)dx=\int_{\Gamma_{N}}g^{T}\left({\tilde{u}}_{t+s}-u_{t}\right)dS_{x}\\ \;+\int_{\Gamma_{t}}n_{t}^{T}\left[\left[p_{re}({\tilde{u}}_{t+s}-u_{t})\right]\right]dS_{x}+sB_{u}\left(s,\left({\tilde{u}}_{t+s},{\tilde{p}}_{t+s}\right),{\tilde{u}}_{t+s}\right)+sB_{p}\left(s,\left({\tilde{u}}_{t+s},{\tilde{p}}_{t+s}\right),{\tilde{p}}_{t+s}-p_{re}\right)\\ +\int_{\Omega_{t}}(\left[Sp_{t-\delta }+\alpha tr\;\varepsilon \left(u_{t-\delta }\right)\right]\left({\tilde{p}}_{t+s}-p_{t}\right)+\left[S\left({\tilde{p}}_{t+s}-p_{t}\right)+\alpha tr\;\varepsilon \left({\tilde{u}}_{t+s}-u_{t}\right)\right]p_{re}+\delta \kappa \nabla {\left({\tilde{p}}_{t+s}-p_{t}\right)}^{T}\nabla p_{re}\\ \;\;-\tau^{0}:\varepsilon \left({\tilde{u}}_{t+s}-u_{t}\right)-2\left[A\varepsilon \left(u_{t}\right):\varepsilon \left({\tilde{u}}_{t+s}-u_{t}\right)+Sp_{t}\left({\tilde{p}}_{t+s}-p_{t}\right)+\delta \kappa \nabla p_{t}^{T}\nabla \left({\tilde{p}}_{t+s}-p_{t}\right)\right])dx. \end{array}$$

On taking the limit as $s_{k}\to 0^{+}$ in equation (92) due to the boundedness (86) and convergences (89) and (90), it follows

(93)
$$\left({\tilde{u}}_{t+s_{k}},{\tilde{p}}_{t+s_{k}}\right)\to \left(u_{t},p_{t}\right)\;\mathrm{strongly}\;\mathrm{in}\;H^{1}{\left(\Omega_{t}\right)}^{d}\times H^{1}\left(\Omega_{t}\right),$$

for elliptic A from equation (16) and positive κ from equation (17).

#### Strong convergence of ${\tilde{\lambda }}_{t+s_{k}}$

Subtracting the reference equation (28) from equation (87) such that

(94)
$$\begin{array}{c} {〈{\tilde{\lambda }}_{t+s}-\lambda_{t},n_{t}^{T}\left[\left[\tilde{v}\right]\right]〉}_{\Gamma_{t}}=-\int_{\Omega_{t}}\left(A\varepsilon \left({\tilde{u}}_{t+s}-u_{t}\right)-\alpha \left({\tilde{p}}_{t+s}-p_{t}\right)I\right):\varepsilon \left(\tilde{v}\right)dx\\ \;+sB_{u}\left(s,\left({\tilde{u}}_{t+s},{\tilde{p}}_{t+s}\right),\tilde{v}\right)-sB_{\lambda }\left(s,{\tilde{\lambda }}_{t+s},\tilde{v}\right), \end{array}$$

dividing it by the norm of $n_{t}^{T}[[\tilde{v}]]$, and taking supremum over $\tilde{v}$, applying the Cauchy–Schwartz inequality by the virtue of the boundedness (86) and (88) and the convergence (93), from equation (94) we conclude that

$${\tilde{\lambda }}_{t+s_{k}}\to \lambda_{t}\;\mathrm{strongly}\;\mathrm{in}\;H_{00}^{1/2}{\left(\Gamma_{t}\right)}^{★}.$$

Together with equation (93), this finishes the proof of the strong convergence (59) in Trait 4.
